# Nutrition, Exercise, and Cognitive Rehabilitation for Dementia Prevention

**DOI:** 10.14789/jmj.JMJ23-0032-R

**Published:** 2024-02-16

**Authors:** TOMOKAZU TAKAKURA

**Affiliations:** 1 Department of Rehabilitation Medicine, Juntendo University Graduate School of Medicine, Tokyo, Japan; 1 Department of Rehabilitation Medicine, Juntendo University Graduate School of Medicine, Tokyo, Japan; 2 Department of Rehabilitation Medicine, Juntendo Tokyo Koto Geriatric Medical Center, Tokyo, Japan; 2 Department of Rehabilitation Medicine, Juntendo Tokyo Koto Geriatric Medical Center, Tokyo, Japan

**Keywords:** dementia, nutrition, physical exercise, cognitive rehabilitation, repetitive transcranial magnetic stimulation

## Abstract

Dementia is one of the most significant global challenges in medical and social care in the 21st century. It affects not only the patients themselves, but also their families, caregivers, and society in general, causing physical, psychological, and socioeconomic effects. As of 2020, there are approximately 6 million people in Japan aged 65 or older with dementia, and this number is expected to increase to around 7 million by 2025, meaning that one out of every five elderly people will have dementia. To prevent the onset and progression of dementia, it is crucial to have a proper understanding of its risks and adopt a healthy lifestyle. Leading an active life from an early stage can also aid in delaying or preventing the onset of dementia. Livingston has identified 12 risks that can lead to dementia, including physical inactivity, smoking, excessive alcohol consumption, air pollution, head injury, social isolation, poor educational history, obesity, hypertension, diabetes, depression, and hearing loss. Modifying one's lifestyle and leading an active life can be crucial in reducing these risks. The Mediterranean diet is gaining attention as a good practice for dementia prevention due to its diversity, richness in omega-3 fatty acids and vitamins. Exercise has been shown to prevent dementia on biological, behavioral, and socio-psychological levels. Repetitive transcranial magnetic stimulation is a non-invasive brain stimulation method that can alter brain plasticity and is being studied for clinical applications as a non-drug therapy for preventing dementia progression.

## Introduction

A super-aging society is one in which the elderly population aged 65 and over accounts for 21% or more of the total population. In Japan, due to the rapid aging of the population, the country has already entered a super-aging society in 2007. According to recent data from the Statistics Bureau of the Ministry of Internal Affairs and Communications, Japan's elderly population aged 65 and over is projected to reach 36.27 million by October 2022. This translates to 29.1% of the total population, representing a 0.3 percentage point increase from the previous year's figure of 28.8%. These figures mark the highest ever recorded for this demographic group. Furthermore, according to the same report, as of October 2022, there will be 19.37 million people aged 75 or older in the late stages of life, accounting for 15.5% of the total population. In comparison with other countries, Japan (29.1%) has the highest percentage of elderly in the total population, followed by Italy (24.1%), Finland (23.3%) Puerto Rico (22.9%), and Portugal (22.9%)^1)^. By 2025, the number of the elderly aged 75 and over is expected to reach 21.8 million, and the number of the elderly aged 65-74 is expected to reach 14.97 million, totaling about 37 million people aged 65 and over. On the other hand, Japan's total population peaked in 2008 and has been declining since then. Therefore, as a result of the relative increase in the elderly and relative decrease in the number of young people due to demographic changes, about one in three of the nation's population will be 65 years of age or older, and about one in five will be 75 years of age or older. The baby boomer generation born between 1947 and 1949 will enter late-elderly status in 2025, marking a significant milestone^2)^. This will have a major impact on society as a whole in terms of social security, medical care, long-term care, and pensions to support the growing number of late-elderly citizens, and it has been likened to the “2025 problem,” and the problem is expected to become even more apparent. To maintain the social security system in the future, it is becoming increasingly important to extend the healthy life expectancy of every citizen.

Dementia is generally defined as a decline in cognitive function beyond what is normally expected with aging. Cognitive decline and psychiatric symptoms of dementia are commonly divided into higher brain dysfunctions such as memory impairment, aphasia, and apraxia, which are considered core symptoms, and behavioral and psychological symptoms of dementia (BPSD) such as delirium, hallucinations, and decreased vitality. Dementia is one of the greatest global challenges in health and social care in the 21st century^3)^. It is estimated that there are currently more than 55 million patients worldwide, with an additional 10 million new cases each year^4)^. According to the Ministry of Health, Labour and Welfare's survey on long-term care insurance certification in Japan, dementia is the leading factor in the need for long-term care^5)^. Dementia has physical, psychological, and socioeconomic effects not only on the patients themselves, but also on their families, caregivers, and society. Taking active steps towards dementia prevention is crucial to maintain not only the health of the patient but also of their family and social circle. This approach can also help in reducing the demand for medical care in society as a whole. As of 2020, there will be approximately 6 million people in Japan aged 65 or older with dementia, and this number is expected to increase to approximately 7 million by 2025, meaning that one out of every five elderly people will have dementia^6)^. With such a rapidly aging population, there is no doubt that the prevention of the onset and progression of dementia is one of the most important and urgent issues in Japan. On a global scale, efforts to understand and prevent dementia are gradually being made, and the number of dementia patients is decreasing in developed countries. To prevent the onset and progression of dementia, it is crucial to have a proper understanding of its risks and adopt a healthy lifestyle. Leading an active life from an early stage can also aid in delaying or preventing the onset of dementia. This article outlines the risks of developing dementia and the non-pharmacologic therapies, such as diet, nutrition, exercise, and cognitive rehabilitation, that are necessary to prevent dementia.

## Causal diseases of dementia

Alzheimer's disease (AD) is the most common causative disease of dementia in Japan at 67.4%, followed by cerebrovascular dementia (CVD) at 18.9%, dementia with Lewy bodies (DLB) at 4.6%, mixed dementia at 4.2%, and frontotemporal dementia (FTLD) at 1.1%^7, 8)^. These dementias are fatal diseases and have a prognosis of 10 years or less, depending on the age of onset^9, 10)^.

On the other hand, there is a type of dementia that can be treated, which is referred to as “treatable dementia”^11)^. Treatable dementia can occur at a young age and often has a more acute course than AD and other degenerative diseases. It is important to note that there are several types of dementia that can be treated effectively. These include endocrine disorders such as hypothyroidism, essential vitamin deficiencies such as vitamin B1, vitamin B12, and folate deficiencies, neurosyphilis, fungal infections, central nervous system infections such as encephalitis and meningitis, chronic subdural hematoma, normal pressure hydrocephalus, and nonconvulsive status epilepticus. These treatable dementias can occur even in patients already diagnosed with AD, CVD, DLB, etc. However, if these diagnoses have already been made, any exacerbation of symptoms tends to be dismissed as a symptom progression of this disease, and then cognitive declines to be overlooked without an opportunity to be re-examined.

Physicians and healthcare professionals who treat patients with dementia must always take into account the impact of multiple factors. Rather than attributing fluctuations in mental symptoms to a single disease, they should approach diagnosis and treatment flexibly. This should be done considering both progressive dementia and treatable dementia, and adapting to changes in the patient's condition. Furthermore, the human “mind” and “body” are closely linked, and cognitive functions, mental states, and physical functions interact with each other. In particular, as represented by the term “brain-gut correlation”, gastrointestinal tract functions, autonomic nervous system functions, and brain functions interact with each other. Therefore, it must be recognized that in patients with dementia, cognitive and mental status fluctuate due to various physical complications such as infections, pain from intra-abdominal organ diseases, falls, and fractures, and that dementia can easily become severe. It is important to avoid polypharmacy and cognitive decline when treating individual diseases. When different specialists focus on treating specific organs, they may overlook the effects of drug administration, resulting in a vicious cycle of treating physical diseases and cognitive decline. Many patients who develop dementia are elderly and are prone to unanticipated side effects from drugs. As dementia is a progressive systemic disease that starts with cognitive decline, it is important to conduct a thorough assessment of the entire body, including cognitive function, internal organs, and the musculoskeletal system, and to administer the minimal drug therapy required. Therefore, it is important to select treatment and care options from a bird's- eye viewpoint based on a thorough understanding of the characteristics and limitations of both pharmacological and non-pharmacological therapies.

## Global trends in the increase and decrease of dementia patients

The world population is getting older, which is resulting in a rise in the number of people diagnosed with dementia. As per the estimates of 2019, the number of dementia patients is around 57.4 million^12)^. It is expected to increase to 152.8 million, almost triple the current number, by 2050^4)^. The number of dementia patients is expected to increase in line with population growth, particularly in sub- Saharan Eastern Africa, North Africa, and the Middle East. In East Asia, on the other hand, the aging of the population is expected to be a major factor in the increase in the number of dementia patients^12)^. However, it has been reported that in high-income countries in the United States and Europe, the prevalence of dementia has been declining in recent years^13-15)^, the incidence of dementia has also been reported to be declining in recent years^16-18)^. In a study conducted by Langa et al. from the University of Michigan, USA, the prevalence of dementia was compared between the years 2000 and 2012 in the US. The study revealed that there was a significant decrease in the prevalence of dementia during this period, and that this reduction was partly attributed to an increase in the number of years of education^14)^.

However, the number of dementia patients in Japan has been increasing in recent years, and the situation differs from that in other developed countries^19)^. The increase in the number of elderly people in Japan has outpaced that of other developed countries^20)^ then the aging of the population in Japan is much higher than in other Western countries. From the well-known Hisayama Town study, a cohort study of lifestyle-related diseases, the prevalence of dementia patients is on the rise^21)^, and reports show that cerebrovascular dementia is decreasing, while tauopathies like AD and SD-NFT are increasing with society's aging^22, 23)^.

## Current status of pharmacotherapy for dementia diseases

AD is the most common form of dementia, but as of August 2023, no drug can cure AD. In Japan, four drugs have been approved for the treatment of dementia: three cholinesterase inhibitors (ChEIs) and one NMDA receptor antagonist, but these are all symptomatic therapies^24)^. The choline hypothesis has been proposed to explain cognitive dysfunction in AD, which is thought to be caused by a decline in the function of the acetylcholine (ACh) system in the central nervous system^25-27)^. Three drugs currently approved for the treatment of AD, donepezil, galantamine, and rivastigmine, are cholinesterase inhibitors (ChEI) that were developed to inhibit the degradation of ACh^28)^. In AD, the NMDA receptor (N-methyl-D-aspartate receptor: NMDAR), one of the glutamate receptors, is activated excessively, which causes neurotoxicity and cognitive dysfunction^29)^. Memantine was developed for the treatment of AD based on the hypothesis of glutamate-induced neurotoxicity and is thought to antagonize NMDAR, inhibit excessive glutamate stimulation, improve synaptic transmission, and inhibit neuronal cell loss^30)^. ChEI affects both peripheral and central cholinesterase, causing side effects such as gastrointestinal and cardiovascular symptoms, frequent urination, muscle spasms, nightmares, and insomnia due to excess Ach^24)^. It is important to note that while ChEI may improve motivation and activity, it also increases irritability and can be challenging to differentiate from delirium. It is important to note that while ChEI may improve motivation and activity, it also increases irritability and can be challenging to differentiate from delirium. Memantine also causes side effects such as somnolence, dizziness, confusion, headache, constipation, and incontinence.

A survey conducted between 2015 and 2016 in Japan found that almost half of those prescribed antidementia drugs were 85 years or older. This is surprising because 17% of the elderly aged 85 years or older were prescribed some of these anti-dementia drugs^31)^. The target population for these anti-dementia drugs in the clinical trial phase was 85 years of age or younger, and the current situation in prescribing is becoming divergent from the subjects targeted in the clinical trials. The prescribing of anti-dementia drugs to super-aged patients must be approached with greater caution due to insufficient efficacy and safety, and potential adverse events. These drugs are only intended to alleviate the psychiatric symptoms of dementia, not to treat the disease itself. Because the effects of these dementia medications are difficult to understand and side effects are difficult to detect, behavioral and psychological changes due to side effects can easily be misinterpreted as worsening BPSD symptoms or dementia symptoms. In France, as of August 2018, the four dementia drugs mentioned above have been excluded from insurance coverage due to their potentially serious side effects, while their effects on patients' behavioral disturbances, quality of life, and mortality have not been established. The government is shifting its policy to focus on long-term care and comprehensive community care rather than these drugs whose usefulness is not yet certain.

Disease-modifying drugs have long been under development as therapeutics for the fundamental treatment of dementia, but many of them have failed in the clinical trial stage.

The U.S. FDA approved the use of lecanemab in July 2023. Lecanemab is an antibody drug that is based on the amyloid hypothesis. It is designed to remove amyloid-β accumulation in the brains of patients with Alzheimer's disease. Lecanemab is the first drug that is expected to modify the disease course of Alzheimer's disease. It is expected that lecanemab will be approved in Japan shortly. However, before administration of lecanemab, a positron emission tomography (PET) scan or spinal fluid analysis must be performed to confirm the presence of amyloid-β^32)^. This creates a hurdle for patients to choose whether they can afford an expensive PET scan or an invasive CSF test before they start receiving lecanemab. In addition, cerebral edema and microhemorrhages, known as amyloid-related imaging abnormalities (ARIA)^33, 34)^, have been reported as serious side effects of this drug. Regular MRI scans are required to monitor these side effects^32)^. In addition, testing for APOE4 gene carriage is necessary for some APOE4 gene carriers who are at an increased risk of developing ARIA^33)^. Individuals who are prescribed anticoagulants for the prevention of cardiac disease or stroke are at a greater risk of experiencing cerebral hemorrhage^32)^. The administration of lecanemab concurrently with anticoagulants may intensify the likelihood of adverse effects, posing a challenge when considering this medication for patients who have a history of stroke or ischemic heart disease. The price of lecanemab in the U.S. is set at $26,500 per year^35)^, making it currently a very expensive treatment. Although the improvement of dementia symptoms by this treatment is expected to have an economic benefit by reducing the burden of caregiving and financial burden on those around the patient, there are concerns about the increased burden on medical resources, such as twice-monthly intravenous infusions and periodic MRI scans. It remains uncertain whether the benefits of disease- modifying drugs outweigh the risks of side effects and the expenses associated with medical resources. Additionally, it may take some time before these drugs become readily accessible and commonly used as a treatment option worldwide. Although there are high expectations for the emergence of disease-modifying drugs, their indication is currently limited to MCI or mild AD, and they are expected to be effective as secondary or tertiary prevention after such a diagnosis has been made. In the current situation where we have to deal with the increasing number of dementia patients on a global scale, efforts for primary and secondary prevention, particularly through non-pharmacologic interventions like lifestyle modifications, are critical.

## Stages of progression from normal to dementia

The process of progression from normal to dementia is classified into four stages. The first stage is called subjective cognitive decline (SCD)^36, 37)^, in which the patient has only subjective symptoms of forgetfulness, followed by mild cognitive impairment (MCI)^38, 39)^, in which the patient has objectively mild cognitive impairment but is still independent in daily living, and finally dementia when the symptoms progress further and daily living becomes difficult to be independent. It is estimated that 5-20% of MCI patients will develop dementia in a year^39)^. MCI has also been reported to revert to normal cognitive function^40, 41)^, early diagnosis and prevention of dementia in the MCI stage is extremely important.

## Dementia symptoms and stages of progression

Dementia is commonly associated with cognitive function and mental symptoms. However, it is a degenerative brain disease that progressively causes a variety of brain dysfunctions. It can lead to a decline in motor abilities such as walking, standing, and balance^42, 43)^. Additionally, it can also worsen swallowing and eating functions^44-46)^. Mental functions gradually deteriorate, leading to delirium and psychomotor agitation, eventual loss of facial expressions, communication difficulty, inaction, self- enclosure, immobility, and muteness. As the disease progresses, patients may experience complications such as malnutrition, aspiration pneumonia, pyelonephritis, sepsis, injuries from falls, and ultimately death.

In the case of AD, the main initial symptom is memory impairment, such as the inability to remember the names of people one knows well. After experiencing memory impairment, individuals may develop symptoms such as executive dysfunction, disorientation, apraxia, and unconsciousness which can hinder their ability to perform instrumental activities of daily living. In the middle and later stages, basic ADLs are impaired and the patient requires nursing care. During the course of the disease, individuals may develop symptoms such as anxiety, depression, insomnia, agitation, irritability, paranoia, wandering, hallucinations, and delusions that may require pharmacotherapy. The progression of AD is assessed by the Functional Assessment Staging of Alzheimer's Disease (FAST) developed by Reisberg in 1984 and is used worldwide to assess the progression of AD^47, 48)^. FAST is composed of 7 stages, 5 sub-stages for Stage 6 and 6 stages for Stage 7, and a total of 16 stages. Reisberg emphasizes the need to watch for the worsening of disease due to treatable factors, including electrolyte abnormalities and stroke complications, when symptoms progress more rapidly than the typical course shown in FAST. (Table 1)

**Table 1:** The Functional Assessment Staging of Alzheimer's Disease (FAST)

Stage	Clinical diagnosis	Characteristics
1	Normal adult	No difficulty either subjectively or objectively
2	Normal aged adult	Complains of forgetting the location of objects—subjective word-finding difficulties
3	Compatible with incipient AD	Decreased job functioning evident to co-workers. Difficulty in traveling to new locations. Decreased organizational capacity
4	Mild AD	Decreased ability to perform complex tasks (e.g., planning dinner for guests, handling. personal finances, difficulty marketing, etc.)
5	Moderate AD	Requires assistance in choosing proper clothing to wear for the day, season, or occasion (e.g., a patient may wear the same clothing repeatedly unless supervised)
6a	Moderately severe AD	Improperly putting on clothes without assistance or prompting (e.g., may put street clothes on overnight clothes, put shoes on wrong feet, or have difficulty buttoning clothing) occasionally or more frequently over the past weeks
6b		Unable to bathe properly (e.g., difficulty adjusting bathwater temp.) occasionally or more frequently over the past weeks
6c		Inability to handle mechanics of toileting (e.g., forgets to flush the toilet, does not wipe properly or properly dispose of toilet tissue) occasionally or more frequently over the past weeks
6d		Urinary incontinence occasionally or more frequently over the past weeks
6e		Fecal incontinence occasionally or more frequently over the past weeks
7a	Severe AD	Ability to speak limited to approximately a half-dozen different intelligible words or fewer in an average day or the course of an intensive interview
7b		Speech ability is limited to using a single intelligible word on an average day or in an intensive interview (the person may repeat the word over and over)
7c		Ambulatory ability is lost (cannot walk without personal assistance)
7d		Cannot sit up without assistance
7e		Loss of ability to smile
7f		Loss of ability to hold head up independently

Adapted from Reisberg B (1986) Geriatrics, 41: 30-46.

## Three phases in dementia prevention

There are three phases in dementia prevention^49)^. Primary prevention is to prevent the onset of dementia from a healthy and asymptomatic state. Secondary prevention is to delay the onset of dementia through early detection and treatment. Tertiary prevention is to delay the progression of symptoms after the onset of dementia. In the primary and secondary prevention stages, non- drug therapy and lifestyle modification are most important. In the tertiary prevention stage, pharmacotherapy may be necessary for BPSD symptoms such as hypoactivity, apathy, and psychomotor agitation; however, it should only be used as part of an overall treatment plan that also includes non-pharmacological interventions.

Even if a patient has already been diagnosed with a dementia disease such as AD or DLB, complications of “treatable dementia” such as hydrocephalus, chronic subdural hematoma, endocrine abnormalities, and essential vitamin deficiencies may exacerbate dementia symptoms. In addition, as patients age, they are more likely to suffer head trauma from easy falls due to the progression of frailty, and polypharmacy due to the increased use of medications to treat increased medical complications also contributes to the progression of frailty. For these various reasons, the overlap of various diseases and conditions can lead to further cognitive decline. Older people suffering from dementia are at increased risk of malnutrition due to various nutritional problems^50)^. In particular, after the onset of dementia, it is not uncommon for nutritional disorders to develop as a result of decreased food intake due to decreased food recognition and interest, impaired chewing and eating function, and other complications such as vitamin B1 deficiency (Wernicke-Korsakoff encephalopathy), vitamin B12 deficiency, folate deficiency, and hypoglycemic encephalopathy associated with decreased food intake. Complications associated with low dietary intake can worsen symptoms of dementia. Poor nutritional status increased BPSD, especially verbal aggressiveness/emotional disinhibition, in those with MCI and early-stage AD. Patients with MCI and early-stage AD need to be assessed for nutritional status from early on, at the onset of mild cognitive decline, and require intervention to prevent worsening of BPSD^51)^.

Therefore, even after the diagnosis of dementia is confirmed, it is important to continue periodic imaging with head CT, MRI, and periodic blood sampling for evaluation of thyroid hormone abnormalities and vitamin deficiency as tertiary prevention after the onset of dementia.

## Risk factors related to the development of dementia

**Figure 1:**
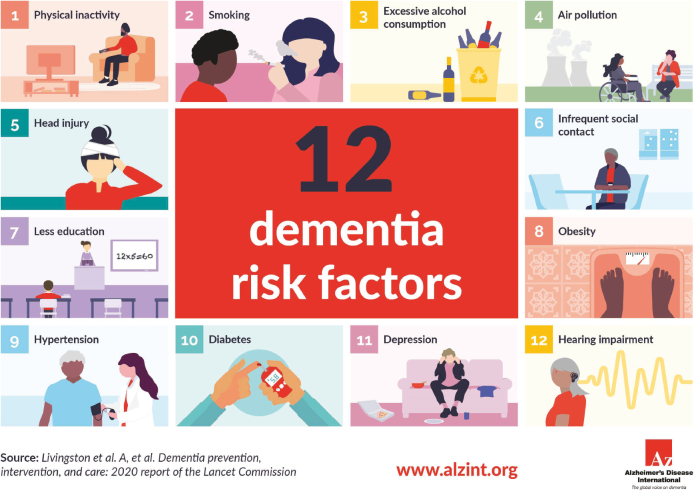
Twelve potentially modifiable risk factors presented as infographic by Alzheimer's Disease International (ADI), based on Livingston's report: Dementia prevention, intervention, and care: 2020 report of the Lancet Commission https://www.alzint.org/about/risk-factors-risk-reduction/

Sixty percent of the risks for dementia are difficult to modify, while 40% are considered modifiable. The “12 modifiable risks” proposed by Livingston, University of London, include physical inactivity, smoking, excessive alcohol consumption, air pollution, head trauma, social isolation, poor educational history, obesity, hypertension, diabetes, depression, and hearing loss(Figure 1). Maintaining an active and social lifestyle is crucial in preventing dementia. It is recommended to focus on dementia prevention from an early age, but it's never too late to take steps to prevent it. As the saying goes, “It is never too early or too late to be greedy in preventing dementia.” The report highlights the importance of engaging in activities that prevent dementia, regardless of age^52)^.

## Diabetes increases risk of dementia

Several epidemiologic studies have shown that type 2 diabetes decreases cognitive function and increases the incidence of dementia; the Rotterdam Study reported that type 2 diabetes increases the risk of developing dementia and AD, especially during insulin therapy^53)^. The Hisayama study also reported that type 2 diabetes significantly increased both AD and VaD^54)^. The pathogenesis of cognitive dysfunction due to type 2 diabetes includes tau phosphorylation due to increased insulin resistance, neuronal and synaptic damage via amyloid-β accumulation, and vascular damage. Other postulated mechanisms of cognitive dysfunction due to type 2 diabetes include neuropathy and vascular damage mediated by increased amyloid-β accumulation, advanced glycation end-products (AGEs) accumulation due to hyperglycemia, inflammation, and increased reactive oxygen species (ROS) production^55)^.

Postprandial hyperglycemia and diurnal fluctuations in blood glucose are risks for developing dementia. In diabetes mellitus, the hazard ratio for developing dementia was 1.40 times higher for postprandial blood glucose levels of 190 mg/dL compared to 160 mg/dL^56)^. Therefore, it is important to limit carbohydrate intake, consume foods with a low glycemic index (GI) (brown rice and other unrefined grains rather than white rice), increase the number of chews and eat as slowly as possible, and consume vegetables before carbohydrates to reduce the rise in blood glucose levels (meal order). It is important to maintain a diet that does not cause hyperinsulinemia. Hypoglycemia tends to cause irreversible changes in the brain, and it has been reported that the hazard ratio for developing dementia increases from 1.26 for a single hypoglycemic attack to 1.80 for two attacks and 1.94 for three or more attacks^57)^. The frequency of hypoglycemic attacks is also high in patients after the onset of dementia and is related to the fact that hypoglycemic attack symptoms are not easily recognized, meals are more likely to be irregular, and patients are more likely to be undernourished. Especially in elderly patients with frailty, hypoglycemia can easily lead to falls and fractures, creating a vicious cycle.

## Diet and nutritional management for dementia prevention

**Figure 2:**
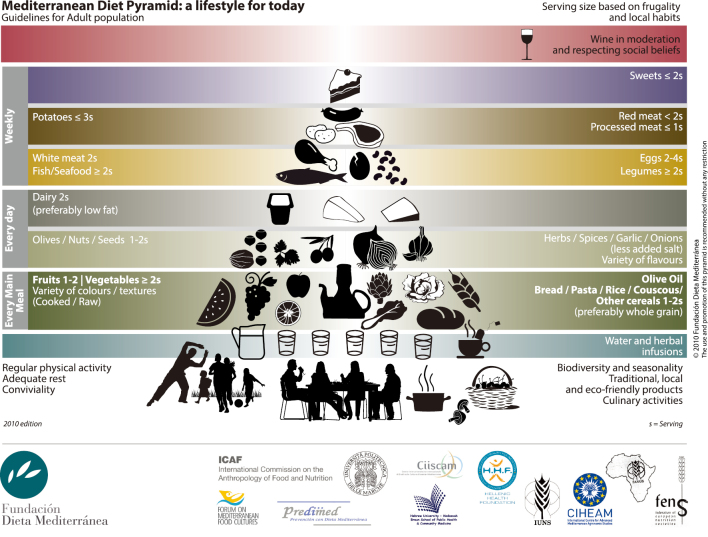
Mediterranean diet pyramid Retrieved from FUNDACIÓN DIETA MEDITERRÁNEA website: https://dietamediterranea.com/nutricion-saludable-ejercicio-fisico/#piramide

In recent years, the Mediterranean diet has been attracting attention as a good dietary practice for dementia prevention^52, 58^^-^^62)^. The Mediterranean diet is a dietary habit of Mediterranean countries such as Italy, Spain, and Greece, characterized by abundant use of fresh fruits and vegetables, more fish than dairy products and meat, olive oil, nuts, legumes, and often unrefined grains such as whole grains. It is also characterized by the use of whole grains and other unrefined cereals. They also drink moderate amounts of red wine with their meals and can be characterized as having a very diverse eating style. (Figure 2) Adherence to the Mediterranean diet was associated with a 20% lower risk of dementia overall in a Mediterranean study involving over 16,000 middle-aged and elderly participants followed up for over 20 years. Associations were stronger for non-AD dementia in women and for AD in men, and among participants with lower education^63)^. There are significant geographical and historical differences between Japan and the Mediterranean countries in Europe, which result in unique food cultures. As a result, it may be challenging to replicate the Mediterranean diet entirely. However, there are still some valuable points that we can learn from it. Compared to the Mediterranean diet, the traditional Japanese diet has a higher carbohydrate intake due to the consumption of rice. Therefore, to build a highly diversified diet based on the Japanese diet, it is recommended that a balanced diet be maintained by actively consuming fish, shellfish, vegetables, fruits, nuts, and legumes, and by moderating the intake of white rice as a carbohydrate and including millet and unrefined grains with low GI values.

Consuming fish is a great way to get protein and omega-3 fatty acids, which are crucial for preventing dementia. Shellfish is a great source of essential minerals such as calcium, iron, zinc, magnesium, and potassium, as well as vitamin B12. Vegetables and fruits are packed with vitamins C, carotene, vitamin E, and folic acid. Nuts are also an excellent source of calcium, magnesium, and dietary fiber. Beans provide protein and vitamin B1, while unrefined grains offer carbohydrates, vitamin B1, minerals, and dietary fiber. Additionally, olive oil is rich in oleic acid, and red wine contains polyphenols. Consuming a diverse range of nutrient-rich foods is essential in preventing dementia. Nutritional management is important for preventing dementia progression, even after onset.

Patients with dementia often experience various eating behavior abnormalities^64)^, such as undernutrition due to anorexia, and overnutrition due to overeating. Furthermore, dementia patients have a reduced sense of taste and tend to prefer sweet and strong-tasting foods^65)^, leading to a diet that is high in carbohydrates and deficient in essential vitamins, minerals, and proteins. This can further exacerbate the disease condition and its symptoms.

## Adequate fatty acid intake for dementia prevention

Fatty acids are divided into four general categories: saturated, monounsaturated, polyunsaturated, and trans fatty acids^66)^. Saturated and trans fatty acids increase the risk of coronary heart disease. Monounsaturated and polyunsaturated fatty acids decrease the risk of coronary heart disease^66)^. The brain is a lipid-rich organ, then the intake of good fats is crucial for the brain. Omega-3 (n-3) fatty acids have been linked to healthy aging throughout life^67)^. The omega-3 fatty acids from fish, eicosapentaenoic acid (EPA), and docosahexaenoic acid (DHA) are associated with fetal development, improved cardiovascular function, and Alzheimer's disease prevention. EPA reduces neutral fat, preventing arteriosclerosis and heart disease^68, 69)^. DHA is a component of nerve cell membranes in the brain^70)^ and is believed to dilate blood vessels^71)^, improve immunity and suppress inflammation^72)^, and aid insulin function^73)^. DHA and EPA are derived mainly from bluefish. Alpha‐linolenic acid (ALA), which is also a representative of ω-3 fatty acids, is contained in plant-derived oils such as linseed oil, perilla oil, perilla sesame oil, and walnut oil^74)^. People who are not fond of fish should actively try to consume such plant-derived ω-3 fatty acids. Since trans fatty acids are contained in foods containing margarine and shortening (cakes, French fries, potato chips, doughnuts, etc.), one should be careful not to eat too much fast food and sweets.

## Vitamin intake and dementia prevention

Insufficient intake of vitamin B1, B12, and folic acid may lead to dementia^75)^. In particular, vitamin B1 deficiency, known as beriberi, has long been a feared national disease, particularly among the Japanese whose staple food is white rice. Even today, vitamin B1 deficiency is potentially quite common, especially among the elderly, and unfortunately, it is still a condition that has a high risk of going undiagnosed and leading to death^76)^. As vitamin B1 deficiency is a potential risk, it is imperative to conduct serum vitamin B1 measurements and administer vitamin B1 supplementation in infusions for patients with impaired consciousness or nutritional disorders. One of the “treatable dementias” caused by vitamin B1 deficiency is Wernicke's encephalopathy, an acute hemorrhagic upper gray matter inflammation described by Carl Wernicke in 1881, classically known as a triad of impaired consciousness, eye movement disorder, and ataxia. However, the triad is rather infrequent, with only about 16% of patients presenting with all three signs^76)^. The sad reality is that during their initial medical examination, more than 80% of patients remain undiagnosed. It's important to note that despite the prevalence of rich diets, vitamin B1 deficiency remains a common issue that can arise due to various factors. These factors include prolonged periods of unbalanced carbohydrate- heavy diets, excessive alcohol consumption, reduced food intake due to loss of appetite, and strenuous exercise. In addition, long-term diuretic therapy for heart failure and leg edema can cause vitamin B1 deficiency^77)^ due to the water-soluble nature of the vitamin B1; however, vitamin B1 measurements are rarely taken during heart failure treatment. Therefore, it is important to educate clinicians involved in emergency room care and heart failure treatment to prevent treatable dementia. To prevent vitamin B1 deficiency, it is advisable to include familiar foods rich in vitamin B1, such as rice bran, pork fillet, seaweed, eel, soybeans, sesame seeds, peanuts, brown rice, and rye bread in the daily diet.

Vitamin B12 deficiency and folate deficiency are known causes of megaloblastic anemia^78, 79)^ and subacute association spinal cord degeneration^80)^, but even without these conditions, they are known to cause cognitive decline^81)^. Folic acid deficiency and associated elevated homocysteine levels are also thought to cause dementia and affective disorders such as depression^82)^. A study on the association between hyperhomocysteinemia and Alzheimer's disease was conducted to investigate the correlation between blood homocysteine levels and Alzheimer's disease over 8 years in 1,092 dementia- free men and women in the United States with an average age of 76 years. 5 µmol per liter of homocysteine in the blood per liter increased the risk of Alzheimer's disease by 40%. The risk of developing dementia or Alzheimer's disease is doubled in the group with the highest blood homocysteine levels compared to the group with the lowest levels, and subjects with persistently elevated blood homocysteine levels are reported to be at the highest risk of dementia^83)^. It has been suggested that folic acid and vitamin B12 supplementation may help prevent mood disorders, Alzheimer's disease, and vascular dementia^84)^. In postgastrectomy/gastrectomy patients, vitamin B12 deficiency occurs due to insufficient secretion of internal factors secreted by the stomach, which is necessary for vitamin B12 absorption. In addition, long-term administration of proton pump inhibitors (PPIs) is known to impair vitamin B12 absorption and cause dementia^85)^. A study examining long-term PPI administration and the development of dementia reported that long-term users who had used PPIs for a cumulative total of more than 4.4 years had a significantly increased risk of developing dementia by 33% compared to those who had never used PPIs. In contrast, the researchers found no significant increased risk among short- to medium-term users of 4.4 years or less^86)^. From the perspective of dementia prevention, it is advisable to include foods containing vitamin B12 and folic acid in the menu daily. Foods rich in vitamin B12 include shellfish such as clams, and laver, anchovies, sweetfish, pork liver, and chicken liver. However, the liver is high in purines, so care should be taken not to consume too much if you have hyperuricemia or gout. Foods rich in folic acid include seaweed, wakame seaweed, chicken liver, pork liver, green tea, soybeans, parsley, kale, and broccoli.

## Exercise to prevent dementia

It has been calculated that about 3% of all dementia cases could be prevented by increasing levels of physical activity^3)^. Both aerobic exercise and resistance training are important for dementia prevention^87, 88)^. The effects of exercise on dementia prevention are estimated to be at the biological level: increased cerebral blood flow due to exercise, increased BDNF and IFG-1, increased brain capacity such as the hippocampus due to improved insulin resistance, behavioral level: improved sleep, decreased fatigue, improved physical activity and physical function, and social psychological level: improved depression, increased self-efficacy, more friends, improved social networks^89)^. A study examining the correlation between physical activity measured with a physical activity meter and brain volume by MRI imaging reported a correlation between prolonged light-intensity physical activity and increased brain volume^90)^.

For aerobic exercise, daily walking is as important as a low-intensity exercise load. It is recommended to use a pedometer, smartphone, or smartwatch to record the number of steps taken and the amount of activity, as this will help maintain motivation. The recommended aerobic exercise for psychiatric symptoms such as cognitive dysfunction and depression is 5,000 steps or more than 3 METs of moderate-intensity activity for at least 7.5 minutes per day^91)^.

In addition to squats, calf raises, and other bodyweight training, dumbbell exercises with dumbbells are recommended for resistance training. Among various types of physical activities including calisthenics, walking, strength exercise, dancing, yoga, aqua exercise, ball games, bicycling, hiking, tai chi, golf, quoits, Japanese croquet, ten-pin bowling, jogging, martial arts, participation in calisthenics significantly reduced the risk of cognitive decline in community-dwelling older Japanese women, indicating that calisthenics may be a useful type of exercise for promoting dementia prevention^92)^.

## Good sleep for dementia prevention

Good sleep, in addition to diet and exercise, has been highlighted as a modifiable factor in dementia prevention^59, 62)^. Accumulating evidence indicates that sleep disturbances contribute to cognitive decline and increase the risk of Alzheimer's dementia by increasing the beta-amyloid burden. Although sleep disturbances are easily thought of as a consequence of Alzheimer's disease, sleep disturbances are a candidate risk factor for Alzheimer's disease. There is a bidirectional relationship between sleep and Alzheimer's disease, and the systemic hyperinflammation induced by sleep disturbances is thought to increase the beta-amyloid burden and promote the etiology of Alzheimer's disease^93)^.

To achieve good sleep, studies have been reported using a variety of sleep interventions for patients with MCI and Alzheimer's disease. Psychotherapeutic approaches such as cognitive behavioral therapy (CBT), melatonin, suvorexant, and continuous positive airway pressure (CPAP) for obstructive sleep apnea (OSA) have shown promise. Acoustic and transcranial stimulation are also assumed to have significant effects on non-REM sleep, but their long-term cognitive and disease- modifying effects are not yet known^94)^.

## NIBS and cognitive rehabilitation for dementia prevention

**Figure 3:**
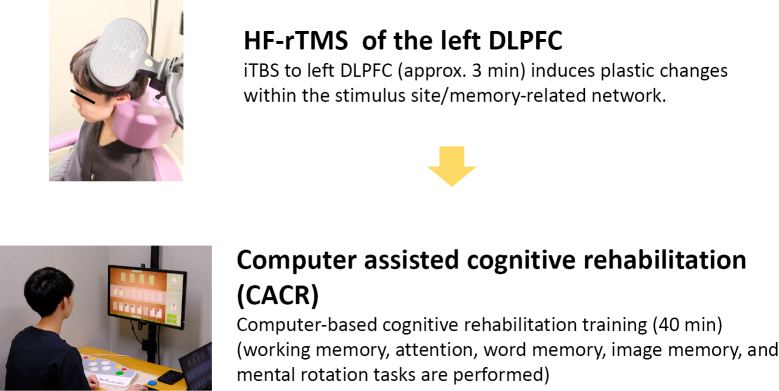
Expectations for rTMS-Cog therapy We are currently implementing a 2-week intensive short-term cognitive rehabilitation treatment program with iTBS to the left DLPFC and CACR for patients with MCI.

We believe that cognitive training is also necessary to treat memory impairment (forgetfulness), which is called the core symptom of dementia. Repetitive transcranial magnetic stimulation (rTMS), a non-invasive brain stimulation (NIBS), is expected to be a non-drug therapy for the prevention of dementia progression. Repetitive transcranial magnetic stimulation (rTMS), a non-pharmacological therapy for the prevention of dementia progression, is being studied for clinical application in Japan and abroad. Although there is no unified consensus on the stimulation site and method, there are many reports using high-frequency stimulation (HF-rTMS) of the left dorsolateral prefrontal cortex (Lt. DLPFC) as in the treatment of depression, and more recently, intermittent theta burst stimulation (iTBS) has been used in some studies. Lazzaro et al. conducted a systematic review of RCTs conducted up to 2020 in AD, MCI, MCI due to Parkinson's disease (PD-MCI), and others^95)^. According to this report, the left dorsolateral prefrontal cortex (DLPFC) was the most frequently stimulated area, followed by bilateral DLPFC, right DLPFC, right DLPFC, low-frequency stimulation of right DLPFC, bilateral DLPFC, and multiple areas such as Broca's area, Wernicke's area, bilateral inferior parietal lobes, and precuneus(Figure 3). This review also concluded that rTMS-Cog therapy with high-frequency stimulation of the left dorsolateral prefrontal cortex (DLPFC) alone and with high-frequency stimulation of multiple regions may improve cognitive function, apathy, memory, and language function in patients with mild (early) AD (Level of Evidence C and B, respectively).

Combined cognitive training (rTMS-Cog therapy) may also be useful to improve cognitive and memory functions in daily life. Computer-assisted cognitive rehabilitation (CACR) is a fun, game-like, repetitive task. Currently, our department is working on cognitive rehabilitation using a combination of rTMS and CACR to improve cognitive and memory functions.

## Summary

In this paper, the key strategies for preventing dementia, which include proper nutrition, exercise, and cognitive rehabilitation were discussed. Dementia prevention is increasingly important as Japan is expected to have around 7 million elderly people with dementia by 2025, which is approximately 1 in 5 elderly people. There are 12 risks identified by Livingston, which include physical inactivity, smoking, excessive alcohol consumption, air pollution, head injury, social isolation, poor educational history, obesity, hypertension, diabetes, depression, and hearing loss. Lifestyle modifications and an active lifestyle are important to reduce these risks. The Mediterranean diet, with its diverse range of foods, has gained attention as a dietary practice for preventing dementia. Exercise has been shown to prevent dementia on biological, behavioral, and socio-psychological levels. rTMS, a noninvasive brain stimulation method, can alter brain plasticity and is being studied for clinical application as a non- drug therapy for preventing dementia progression.

This paper is based on a lecture given at the 51st Juntendo University Open Lecture on June 17, 2023, with some additional information.

## Funding

The author received no financial support for writing this article.

## Author contributions

TT wrote the manuscript. TT read and approved the final manuscript.

## Conflicts of interest statement

The author declares that there are no conflicts of interest.
